# Risk Factors for Hypertension in Polycystic Ovary Syndrome: Evidence from a Retrospective Study

**DOI:** 10.3390/life15091416

**Published:** 2025-09-08

**Authors:** Ralitsa Robeva, Atanaska Elenkova, Georgi Kirilov, Krassimir Kalinov, Sabina Zacharieva

**Affiliations:** 1 USHATE “Acad. Iv. Penchev”, 1000 Sofia, Bulgaria; aelenkova@medfac.mu-sofia.bg (A.E.); zacharieva67@gmail.com (S.Z.); 2 Department of Endocrinology, Faculty of Medicine, Medical University-Sofia, 1000 Sofia, Bulgaria; 3 Scientific Affairs, Medical Center “Comac-Medical”, 1000 Sofia, Bulgaria; krassimir.kalinov@comac-medical.com

**Keywords:** hypertension, blood pressure, PCOS, hyperandrogenic phenotypes, testosterone, DMT2, IFG, IGT

## Abstract

**Background**: Polycystic ovarian syndrome (PCOS) is a common female endocrinopathy, but its interrelations with arterial hypertension (AH) are still debatable. Therefore, the present study aims to explore the risk factors for hypertension in a large group of well-phenotyped women with PCOS. **Methods**: The data of 1047 Bulgarian PCOS patients diagnosed according to Rotterdam criteria in the period 2005–2022 were studied retrospectively. The risk factors for hypertension were estimated in the PCOS women with different phenotypes. **Results**: The prevalence of AH was 17.6% among the PCOS women, with 4.2% of them being on antihypertensive treatment. The AH prevalence was increased in women with the “classic” phenotype compared to others (18.9% vs. 12.9%, *p* = 0.037). The most important risk factors associated with hypertension were the presence of diabetes mellitus type 2 (DMT2), obesity, family history of AH, and age ≥ 30 years (*p* < 0.001). The prevalence of impaired glucose tolerance (IGT) but not impaired fasting glucose was also related to the development of AH. **Conclusions**: The leading independent factors associated with hypertension in PCOS patients are the presence of DMT2, IGT, obesity, family history of hypertension, and age, but not the degree of hyperandrogenism. Population-based studies, including distinct ethnic groups, are needed to reveal the pathophysiology and the optimal clinical management of AH in PCOS.

## 1. Introduction

Polycystic ovarian syndrome (PCOS) is a common female endocrinopathy characterized by variable clinical phenotypes and a myriad of reproductive and metabolic complications [1,2,3]. The first attempt to standardize the diagnosis of PCOS was made by the National Institutes of Health (NIH) in 1990, which considered clinical and/or biochemical hyperandrogenism (HA) and chronic anovulation (CA) as mandatory criteria for the syndrome [4,5]. Fourteen years later, the Rotterdam PCOS Consensus Workshop Group broadened the PCOS definition by identifying HA, CA, and polycystic ovaries (PCO) as equally important criteria [5]. Thus, additional PCOS-phenotypes were described beyond the NIH criteria (HA + CA ± PCOS), e.g., “ovulatory” PCOS (HA + PCO) and “non-androgenic” PCOS (CA + PCO) [4,5,6]. Recently, the Rotterdam criteria were reaffirmed as the cornerstone of PCOS diagnosis [7], thereby emphasizing the phenotypic diversity of the syndrome.

Despite the intensive research focused on PCOS, following the implementation of the standardized Rotterdam criteria, many clinical questions remain unresolved. For instance, the associations between PCOS and hypertension are still a matter of debate [8]. In a large meta-analysis, Wekker et al. report an increased risk for hypertension in PCOS women compared to healthy women [9]. However, Amiri et al. conclude that the odds of hypertension are increased in PCOS women of reproductive age only, but not in older postmenopausal women, compared to controls [10]. Conversely, in a recent meta-analysis focused on late reproductive age, peri-, and postmenopausal women, PCOS has been related to the increased risk for hypertension [11]. Finally, in the most extended follow-up study of PCOS patients and controls up to the age of 80 years, no differences in hypertension and cardiovascular disease prevalence between PCOS and non-PCOS women were observed [12].

On the other hand, the prevalence of hypertension in young PCOS women might vary between 5.5% in Australian and 71.6% in Brazilian cohorts [13,14]. Differences in the reported prevalence of hypertension among PCOS patients may be explained by the heterogeneity of the syndrome and the presence of significant ethnic and racial disparities [10,11,15]. Several studies have investigated the prevalence of hypertension in different PCOS phenotypes, reporting contradictory findings [16,17,18,19]. Moreover, different factors, e.g., obesity, insulin resistance, carbohydrate disturbances, and hyperandrogenia, have been explored as risk determinants for hypertension in PCOS, but the interrelations between them preclude the identification of a leading risk factor associated with the blood pressure increase [13,20,21]. Additionally, ethic differences might influence the risk profile of PCOS, but most PCOS data on hypertension originate from Asian, American, and Northern European populations [11]. Conversely, the data for Eastern European countries are scarce, despite the higher prevalence of PCOS in that region [22]. Therefore, it is not clear whether a similar approach should be used for hypertension screening in all PCOS phenotypes and ethnicities or whether individualized surveillance should be implemented.

Considering the controversies in the literature, the present study aims to explore the risk factors associated with the development of hypertension in a large group of well-phenotyped women with PCOS from Eastern European (Bulgarian) origin. Obtaining more ethnic-specific information about the relationship between different PCOS phenotypes and hypertension may help to individualize and optimize the management of the disease, thus preventing complications.

## 2. Materials and Methods

### 2.1. Study Participants

Medical papers and electronic files of all adult women referred to a single tertiary endocrine department in Sofia (Bulgaria) with hyperandrogenism (ICD-10 E28.1), polycystic ovarian syndrome (ICD-10 E28.2), and ovarian dysfunction (ICD-10 E28.8) between 2005 and 2022 were studied retrospectively. The inclusion criteria for the study were reproductive age (18–44 years), Caucasian (Bulgarian) origin, and diagnosis of PCOS based on two of the three Rotterdam criteria (HA, CA and PCO) [5].

The exclusion criteria were the presence of other diseases causing the clinical symptoms: congenital adrenal hyperplasia, androgen-secreting tumors, Cushing’s syndrome, acromegaly, and prolactinomas, as well as insufficient data in the medical records to establish PCOS diagnosis and phenotype [5]. PCOS phenotyping was based on the clinical symptoms, laboratory, and ultrasound characteristics before oral contraceptives or antihypertensive treatment.

The Rotterdam criteria identify four different PCOS phenotypes: phenotype A (HA + CA + PCO), phenotype B (HA + CA), phenotype C (HA + PCO), and phenotype D (CA + PCO) [5]. The phenotypes A and B are assumed to be “classic” PCOS phenotypes, resembling the narrow NIH criteria. The other two phenotypes have been identified after the implementation of the Rotterdam criteria and are considered subtypes with a lower risk of metabolic complications [5,6]. Therefore, the patients in the investigated group were divided into three similar groups: patients with “classic” PCOS (Phenotype A or B, AB), “ovulatory” (Phenotype C) PCOS, and “non-hyperandrogenic” (Phenotype D) PCOS.

From the initially selected 1130 patients, 83 were excluded because of missing blood pressure values. From the finally selected 1047 patients, 822 [78.5%] were with the “classic” phenotypes A or B (AB), 108 [10.3%] were with the “ovulatory” phenotype C, and 117 [11.2%] were with the “non-hyperandrogenic” phenotype D.

### 2.2. Study Protocol

The Department of Endocrinology used predefined algorithms to take patients’ histories and provide evaluations. Thus, data on anthropometric characteristics (height, weight), blood pressure (BP), the presence of arterial hypertension, and carbohydrate disturbances were collected from medical records and electronic files. Arterial hypertension was established based on the actual recommendations (office systolic BP of ≥140 mmHg or diastolic BP of ≥90 mmHg at the first visit in the clinic) or previously diagnosed hypertension and antihypertensive treatment [23]. Blood pressure was measured twice after a 10 min rest in a sitting position, with the lower blood pressure recorded as in other similar studies [24]. The presence of type 2 diabetes mellitus (DMT2), impaired fasting glucose (IFG), and impaired glucose tolerance (IGT) were estimated according to global recommendations [25,26] based on the previous medical history of the patients or the actual glucose investigations at the first visit in the clinic. Data about hirsutism (modified Ferriman–Galway score ≥ 8), current and former treatment with oral contraceptives, and metformin were recorded. Additionally, information about family history of diabetes and hypertension, as well as smoking (current and former), was extracted from the medical files.

Testosterone (*n* = 977), glucose (*n* = 1013), and insulin (*n* = 702) levels were extracted from the medical data at the first visit in the clinic. The homeostasis model assessment for insulin resistance (HOMA-IR) was calculated using the following formula: HOMA-IR = fasting serum glucose (mmol/l) × fasting serum insulin (μU/mL)/22.5 [27].

Additionally, women were divided into obese (BMI ≥ 30) and nonobese (BMI < 30), as well as “younger” (age < 30 years) and “older” (age ≥ 30 years) as in other studies [3,28]. Biochemical parameters were determined enzymatically by an automatic analyzer (Cobas INTEGRA 400 plus, Hoffmann La Roche, Basel, Switzerland), while testosterone and IRI were evaluated using commercially available RIA and IRMA kits.

### 2.3. Statistical Methods

Categorical variables were described by absolute and relative (percentage) frequencies; testing the null hypothesis was made by Fisher’s exact test and chi-square test. Continuous variables were expressed as arithmetic means and standard deviations (SD), [median]. Due to the heterogeneity and non-normality of the data distribution, non-parametric (Mann–Whitney U and Kruskal-Wallis) tests were used for post hoc hypothesis testing.

Binary logistic regression was used to model the relationship between the output (hypertension) as a dependent variable and prognostic parameters. A model was estimated for the presence of hypertension, including different risk factors as independent variables. The odds ratios with 95% confidence intervals, as well as a forest plot, were shown. For decision-making, a significance level of 5% was used throughout. The SAS^®^ package version 9.4 (SAS Institute Inc., SAS 9.4 Help and Documentation, Cary, NC, USA: SAS Institute Inc., 2015–2022), as well as MedCalc^®^ Statistical Software version 23.2.8 (MedCalc Software Ltd., Ostend, Belgium; https://www.medcalc.org; assessed on 25 August 2025), were used for the calculations and the graphical presentations.

## 3. Results

The main characteristics of the investigated patients (*n* = 1047) according to the phenotypes are presented on Table 1.

The prevalence of arterial hypertension (AH) was 17.6% (*n* = 184) among the PCOS women, with 4.2% (*n* = 44) of them being on antihypertensive treatment. The prevalence of AH was 18.9% in patients with phenotype AB and 13.0% and 12.8% in patients with phenotype C and D, respectively (*p* = 0.114). Women with the “classic” AB phenotype had higher prevalence of hypertension compared to other PCOS women (18.9% vs. 12.9%, *p* = 0.037).

The differences between the hypertensive and non-hypertensive women are shown in Table 2. Hypertensive PCOS patients were older and more obese than other PCOS women. They have more often insulin resistance and carbohydrate disturbances, as well as family history for hypertension compared to non-hypertensive individuals. PCOS patients with hypertension were also more often hirsute, though no significant differences in the testosterone levels between groups were observed.

In the lean PCOS patients (BMI < 25) the presence of hypertension (4.8%) was associated with a higher BMI (22.5 vs. 20.8 kg/m^2^, *p* = 0.008) and a tendency to higher HOMA-IR (2.4 vs. 1.8, *p* = 0.058) but not with age, presence of hirsutism, or testosterone levels (*p* > 0.100 for all). In the obese PCOS women (BMI ≥ 30), the presence of hypertension (33.4%) was associated with older age (27.0 vs. 25.0 years, *p* = 0.010), hirsutism (83.6% vs. 69.9%, *p* = 0.007), and higher BMI (36.3 vs. 33.9 kg/m^2^, *p* = <0.001) but not with differences in testosterone or HOMA-IR levels (*p* > 0.100 for all).

**Figure 1:**
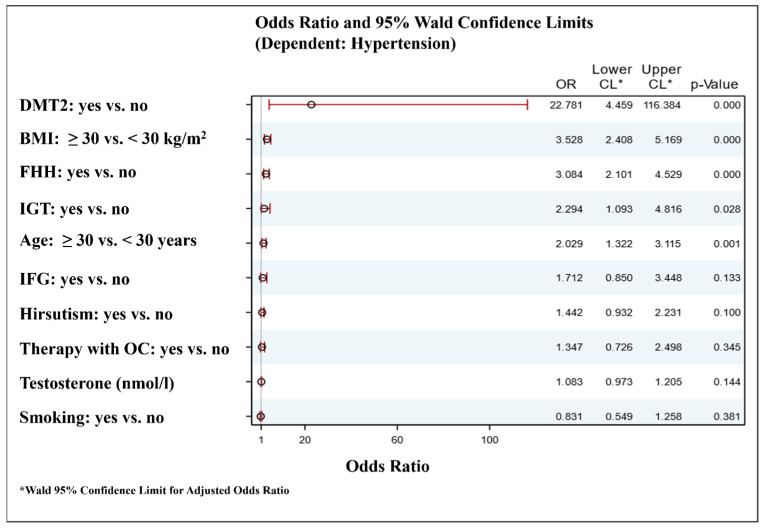
Forest plot for independent factors associated with the hypertension in women with PCOS. DMT2—diabetes mellitus type 2; BMI—body mass index; FHH—family history for hypertension; IGT—impaired glucose tolerance; IFG—impaired fasting glucose; OC—oral contraceptives. Metformin treatment was not included in the model for hypertension risk factors because it might have positive or neutral influence on blood pressure. However, additional inclusion of metformin use as a covariate in the model was not associated with significant differences.

Logistic regression analyses were performed in order to find the main predictors of hypertension among the investigated PCOS women. The model included well-known as well as assumed based on the current data (Table 2) risk factors for hypertension (age, smoking, presence of obesity, carbohydrate disturbances (DMT2, IFG, IGT), family history for hypertension, presence of hirsutism, oral contraceptives use, and testosterone levels). The results showed that age ≥ 30 years, presence of DMT2, IGT, family history for hypertension, and obesity were the most important factors associated with the development of hypertension among the PCOS women (Figure 1).

## 4. Discussion

Our data reveal that the prevalence of arterial hypertension (AH) among a large, well-phenotyped cohort of young Eastern European (Bulgarian) women with PCOS consulted in a tertiary endocrine center was 17.6%. The “classic” phenotype women showed an approximately 50% increase in AH prevalence compared to other PCOS phenotypes. The associations between the PCOS phenotypes and hypertension are still contradictory. For instance, Daan et al. investigated a large group of Dutch women and found a higher prevalence of hypertension in HA-PCOS compared to non-HA PCOS (17.6% vs. 11.8%). At the same time, distinct HA phenotypes did not differ in AH frequency [16]. Conversely, a Brazilian study described a significantly higher prevalence of hypertension in women with “classic” PCOS HA phenotypes compared to the “ovulatory” PCOS individuals [17]. On the contrary, two Asian studies did not observe any differences in hypertension prevalence in women with different PCOS phenotypes [18,19], thus emphasizing the need for an ethnic-specific approach in the evaluation of PCOS-associated health risks.

In our study, the PCOS patients with hypertension were older and more obese, with a higher prevalence of carbohydrate disturbances and therefore increased metformin use compared to patients with normal blood pressure. They were also more hirsute and had higher indices of insulin resistance compared to non-hypertensive women, while no differences in testosterone levels were established. However, considering all investigated factors, some of which were strongly interrelated, the main predictors of hypertension in the PCOS group were the presence of diabetes mellitus type 2, IGT, obesity, family history of hypertension, and age over 30 years, but not hyperandrogenemia. The odds of being hypertensive were highest for PCOS patients with DMT2. Moreover, the prevalence of DMT2 in non-hypertensive patients was similar to the self-reported one in the typical population of young women from the same ethnic group (0.2% vs. 0.4%) [29], while in the hypertensive PCOS individuals, it was 20-fold higher. Similarly, in a large population-based study of South-Asian women, the prevalence of PCOS and DMT2 was associated with a sixfold increase in the odds of hypertension compared to healthy individuals [28].

Hypertension and DMT2 are tightly related in the common population, with 46% of female patients with newly diagnosed DMT2 being hypertensive [30]. Moreover, in healthy individuals, DMT2 at baseline predicts a threefold increase in hypertension incidence over seven years, independently of sex, age, obesity, and family history of DMT2 [31]. Pathophysiological interrelations between hypertension and DMT2 are based on insulin resistance, disturbances in the renin-angiotensin-aldosterone (RAAS) and sympathetic nervous system functions, mitochondrial impairment leading to oxidative stress, proinflammatory state, dysregulation of glucagon-like peptide signaling, and sodium-glucose cotransporter 2 activity [32]. Similar mechanisms may contribute to the close association between hypertension and DMT2 in women with PCOS [2]. Thus, in the present study, DMT2, IFG, and obesity, but not increased fasting glucose, were independent predictors of hypertension. Increased sympathetic nervous system activity and normetanephrine levels have been described in young PCOS patients [33,34]; thus, the chronic hypersympathetic state might contribute to the increased prevalence of hypertension and DMT2 in middle-aged PCOS women. RAAS dysfunction might be another critical factor, considering the increased plasma renin activity, active renin, angiotensin II, and aldosterone levels in women with PCOS, according to most, though not all, studies [35,36,37]. Recently, Amin et al. investigated the mineralocorticoid receptor gene (NR3C2) variants in 212 Italian families with a high incidence of DMT2. They found several novel NR3C2 polymorphisms associated with an increased risk of PCOS in the diabetes-prone participants [38]. Additionally, polymorphic variants in the aldosterone synthase gene associated with increased aldosterone levels and hypertension have been related to PCOS in Chinese women [39,40,41]. Thus, we could assume that specific genetic variants modulating RAAS might predispose a minor subgroup of PCOS patients to early DMT2 and hypertension development and that the risk factors for AH in the diabetic might differ from those in non-diabetic PCOS individuals.

The increased androgen levels were not independently associated with AH in our common group of PCOS women. Testosterone levels correlate directly to the blood pressure values in women with PCOS [42,43]; however, studies provide contradictory findings about differences in hypertension prevalence between hyperandrogenic and normoandrogenic PCOS patients [24,44,45]. Animal studies have revealed complicated associations between androgens and hypertension in PCOS models [46]. Dihydrotestosterone (DHT) administration in female rats increases blood pressure levels and induces a PCOS-like phenotype. After discontinuation of DHT treatment, androgens decrease; however, blood pressure levels remain significantly elevated due to persisting intrarenal RAAS activation and an increase in renal and adipose tissue androgen receptors [47]. Similarly, a recent longitudinal study of PCOS women has revealed that hyperandrogenic patients who remain hyperandrogenic and hyperandrogenic individuals who transition to a normoandrogenic state after 15 years of follow-up have significantly higher hypertension prevalence than women who maintain normal androgens during the same period [48]. Thus, further studies on lifelong changes in androgens and RAAS might help explain the pathophysiology of hypertension in PCOS.

Age is a well-known risk factor for increased blood pressure in PCOS women, while the role of obesity is somewhat contradictory [3,13,21,24,28,49]. According to Joham et al., BMI is independently associated with hypertension in healthy women but not in PCOS patients [13]. Conversely, both age and obesity were significant independent factors associated with hypertension in our PCOS group.

Additionally, familial hypertension but not a family history of DMT2 was found to be a significant predictor of increased blood pressure in our patients. In the common population, familial history of AH is an essential risk factor for hypertension independent of obesity [49,50]; however, in a small study of PCOS women, Sahli et al. did not find associations between family history of AH and AH [51]. According to Cheng et al., only paternal, but not maternal, familial AH and DMT2 are significant risk factors for PCOS metabolic disturbances, but the influence on hypertension was not described [52]. Opposite to our results, a family history of DMT2 has been related to blood pressure levels in Austrian women with PCOS [53]. Thus, further studies are needed to evaluate the role of paternal or maternal DMT2 and AH in developing hypertension in PCOS offspring.

The primary strength of the present study is the large number of well-phenotyped PCOS patients investigated in a single tertiary center. However, it has several limitations, including its retrospective design and the absence of ambulatory 24 h blood pressure monitoring. Thus, neither “white-coat” nor “masked” hypertension could be excluded. Additionally, the phenotype distribution of PCOS patients in clinical settings has been shown to differ from that in the general unselected PCOS population, with obese and phenotype-A patients being more often referred for medical investigations [6,54], which could bias the results. Furthermore, our study could not reveal causal relationships or underlying pathophysiological processes; thus, further prospective studies in different ethnic groups are needed to elucidate the molecular foundations of increased hypertension prevalence in some PCOS subgroups.

In conclusion, our study shows that the leading independent factors associated with the development of hypertension in patients are DMT2, IGT, age, obesity, and family history of hypertension, but not testosterone levels. Thus, our data suggest that obese PCOS patients, PCOS women with first-degree hypertensive relatives, and those with IGT or DMT2, irrespective of weight, should be monitored for hypertension more often than the common PCOS population, where annual blood pressure measurement is recommended [7]. Thus, these specific PCOS groups may need blood pressure follow-up twice yearly and closer follow-up in case of oral contraceptive treatment. Moreover, they might benefit from regular ambulatory 24 h blood pressure monitoring and sleep apnea investigations. Lifestyle interventions could help reduce blood pressure and weight gain and restore fertility; thus, they should be considered crucial for PCOS patients at high risk of hypertension.

It is important to emphasize that a minority of PCOS patients with AH who should have been treated according to current guidelines [23] receive or adhere to anti-hypertensive treatment (lifestyle changes or medical treatment), a tendency also observed in other European countries [55]. Therefore, more efforts are required to implement the developed clinical recommendations in real-world endocrinological and gynecological practice to improve cardiovascular health in women with PCOS.

## Figures and Tables

**Table 1:** Main characteristics of the investigated patients. BMI—body mass index; BP—blood pressure; DMT2—diabetes mellitus type 2; IGT—impaired glucose tolerance; IFG—impaired fasting glucose; OC—oral contraceptives; HOMA-IR—homeostasis model assessment for insulin resistance. Current use is defined as treatment in the last three months. Data are presented as mean ± standard deviation [median] or as number (%).

Parameter	AB Phenotype *n* = 822 [78.5%]	C Phenotype *n* = 108 [10.3%]	D Phenotype *n* = 117 [11.2%]	*p*	All PCOS Women *n* = 1047
Age (years)	25.35 ± 5.44 [24.00]	25.67 ± 5.11 [25.50]	24.91 ± 4.96 [24.0]	0.523	25.33 ± 5.35 [24.00]
**BMI (kg/m^2^)**	**27.48 ± 7.54** **[26.17]**	**25.82 ± 5.96** **[25.00]**	**26.20 ± 7.74** **[23.99]**	**0.029**	27.17 ± 7.44 [25.67]
**Systolic BP (mmHg)**	**114.37 ± 13.80** **[114.00]**	**112.17 + 12.90** **[110.00]**	**109.35 ± 13.97** **[110.00]**	**0.002**	113.59 ± 13.82 [110.00]
**Diastolic BP (mmHg)**	**74.76 ± 9.63** **[75.00]**	**73.73 ± 9.61** **[72.5]**	**71.80 ± 9.14** **[70.00]**	**0.005**	74.32 ± 9.61 [70.00]
Smoker (current or former)	240 (29.2%)	26 (24.1%)	33 (28.2%)	0.530	299 (28.6%)
Family history for DMT2	299 (36.4%)	41 (38.0%)	41 (35.0%)	0.902	381 (36.4%)
Family history for AH	206 (25.1%)	32 (29.6%)	29 (24.8%)	0.581	267 (25.5%)
**Hirsutism**	**625 (76.0%)**	**88 (81.5%)**	**0 (0%)**	**<0.001**	713 (68.1%)
**Testosterone (nmol/L)**	**3.82 ± 1.77** **[3.70]**	**3.52 ± 1.71** **[3.35]**	**2.15 ± 0.90** **[2.17]**	**<0.001**	3.63 ± 1.77 [3.50]
HOMA-IR	**3.29 ± 2.53** **[2.54]**	**2.64 ± 1.79** **[2.19]**	**2.69 ± 1.86** **[2.20]**	**0.035**	3.16 ± 2.40 [2.44]
IFG	45 (5.5%)	3 (2.8%)	7 (6.0%)	0.464	55 (5.3%)
IGT	42 (5.1%)	3 (2.8%)	3 (2.6%)	0.299	48 (4.6%)
DMT2	15 (1.8%)	0 (0%)	3 (2.6%)	0.296	18 (1.7%)
Metformin (current use)	170 (20.7%)	21 (19.4%)	21 (17.9%)	0.770	212 (20.2%)
OC–(current use)	90 (10.9%)	8 (7.4%)	19 (16.2%)	0.100	117 (11.2%)

**Table 2:** Differences between non-hypertensive and hypertensive PCOS women. BMI—body mass index; AH—arterial hypertension; DMT2—diabetes mellitus type 2; IGT—impaired glucose tolerance; IFG—impaired fasting glucose; OC—oral contraceptives; HOMA-IR—homeostasis model assessment for insulin resistance. Current use is defined as treatment in the last three months. Data are presented as mean (SD), [median], or as number (%).

Parameter	Non-Hypertensive PCOS Women (*n* = 863)	Hypertensive PCOS Women (*n* = 184)	*p*-Value
**Age (years)**	**24.9 (5.0)** **[24.0]**	**27.5 (6.4)** **[27.0]**	**<0.001**
**BMI (kg/m^2^)**	**25.9 (6.8)** **[24.2]**	**33.0 (7.3)** **[31.8]**	**<0.001**
Smoker (current or former)	237 (27.5%)	62 (33.7%)	0.105
Family history for DMT2	303 (35.1%)	78 (42.4%)	0.064
**Family history for AH**	**182 (21.1%)**	**85 (46.2%)**	**<0.001**
Testosterone (nmol/L)	3.6 (1.7) [3.5]	3.9 (1.9) [3.6]	0.096
**Hirsutism**	**570 (66.0%)**	**143 (77.7%)**	**0.002**
**HOMA-IR**	**2.9 (2.1)** **[2.2]**	**4.6 (3.2)** **[3.7]**	**<0.001**
**IGT**	**28 (3.2%)**	**20 (10.9%)**	**<0.001**
**IFG**	**38 (4.4%)**	**17 (9.2%)**	**0.016**
**DMT2**	**2 (0.2%)**	**16 (8.7%)**	**<0.001**
**Metformin (current use)**	**161 (18.7%)**	**51 (27.7%)**	**0.008**
OC (current use)	97 (11.2%)	20 (10.9%)	1.000

## Data Availability

Data are available from the corresponding author after reasonable request and with permission from the local authorities.
